# A Systematic Review of the Various Effect of Arsenic on Glutathione Synthesis In Vitro and In Vivo

**DOI:** 10.1155/2020/9414196

**Published:** 2020-07-28

**Authors:** Shanshan Ran, Jiaqing Liu, Shugang Li

**Affiliations:** ^1^Department of Public Health, School of Medicine, Shihezi University, Xinjiang, China; ^2^School of Public Health, Capital Medical University, Beijing, China

## Abstract

**Background:**

Arsenic is a toxic metalloid widely present in nature, and arsenic poisoning in drinking water is a serious global public problem. Glutathione is an important reducing agent that inhibits arsenic-induced oxidative stress and participates in arsenic methylation metabolism. Therefore, glutathione plays an important role in regulating arsenic toxicity. In recent years, a large number of studies have shown that arsenic can regulate glutathione synthesis in many ways, but there are many contradictions in the research results. At present, the mechanism of the effect of arsenic on glutathione synthesis has not been elucidated.

**Objective:**

We will conduct a meta-analysis to illustrate the effects of arsenic on GSH synthesis precursors Glu, Cys, Gly, and rate-limiting enzyme *γ*-GCS in mammalian models, as well as the regulation of p38/Nrf2 of *γ*-GCS subunit GCLC, and further explore the molecular mechanism of arsenic affecting glutathione synthesis.

**Results:**

This meta-analysis included 30 studies in vivo and 58 studies in vitro, among which in vivo studies showed that arsenic exposure could reduce the contents of GSH (SMD = −2.86, 95% CI (-4.45, -1.27)), Glu (SMD = −1.11, 95% CI (-2.20,-0.02)), and Cys (SMD = −1.48, 95% CI (-2.63, -0.33)), with no statistically significant difference in p38/Nrf2, GCLC, and GCLM. In vitro studies showed that arsenic exposure increased intracellular GSH content (SMD = 1.87, 95% CI (0.18, 3.56)) and promoted the expression of p-p38 (SMD = 4.19, 95% CI (2.34, 6.05)), Nrf2 (SMD = 4.60, 95% CI (2.34, 6.86)), and GCLC (SMD = 1.32, 95% CI (0.23, 2.41)); the p38 inhibitor inhibited the expression of Nrf2 (SMD = −1.27, 95% CI (-2.46, -0.09)) and GCLC (SMD = −5.37, 95% CI (-5.37, -2.20)); siNrf2 inhibited the expression of GCLC, and BSO inhibited the synthesis of GSH. There is a dose-dependent relationship between the effects of exposure on GSH in vitro*. Conclusions*. These indicate the difference between in vivo and in vitro studies of the effect of arsenic on glutathione synthesis. In vivo studies have shown that arsenic exposure can reduce glutamate and cysteine levels and inhibit glutathione synthesis, while in vitro studies have shown that chronic low-dose arsenic exposure can activate the p38/Nrf2 pathway, upregulate GCLC expression, and promote glutathione synthesis.

## 1. Introduction

Arsenic is a kind of toxic metalloid which mostly exists in inorganic or organic form in the environment [1]. Arsenic can be absorbed by the body through skin, drinking water, and other ways and then reach all parts of the body through blood circulation. At present, arsenic poisoning in drinking water has become a global public health problem. Among them, arsenic poisoning in China, India, and other countries is more serious. According to the report, 26 million people in India have been affected by arsenic poisoning in drinking water [2]. A large number of studies have found that arsenic can be metabolized through methylation in the body [3]. However, Pratheeshkumar et al. [4] found that arsenic exposure can induce the production of a large number of reactive oxygen species and NOS and promote consumption of antioxidants such as glutathione, inducing oxidative stress. Epidemiological investigation shows that arsenic exposure is closely related to cardiovascular disease, skin cancer, and liver cancer, in which oxidative stress plays an important role [5, 6].

Glutathione (GSH) is an important redox molecule in cells, which can be used not only as a major antioxidant molecule to relieve arsenic-induced ROS [7] but also as a reductant involved in arsenic metabolism to promote arsenic excretion [8]. Therefore GSH plays an important role in inhibiting arsenism. GSH is composed of three nonessential amino acids, glutamate (Glu), cysteine (Cys), and glycine (Gly), and the synthesis process needs to be carried out under the catalysis of GSH synthesis rate-limiting enzyme *γ*-glutaminecysteine synthetase (*γ*-GCS) [9]. *γ*-GCS is mainly composed of catalytic subunit *γ*-glutamate cysteine ligase catalytic subunit (GCLC) and regulatory subunit *γ*-glutamate cysteine ligase regulatory subunit (GCLM) [10]. A large of studies show that arsenic exposure can regulate the synthesis of GSH through Glu and Cys; arsenic can also regulate the synthesis of GSH through the regulation of GCLC by the p38/Nrf2 pathway [11–14].

However, we found that in a large number of studies, there is a lot of controversy about the regulation of GSH synthesis by arsenic. Hou et al. [15] found that chronic arsenic exposure could increase the expression of p38, which was 1.07-fold higher than that of the control group (*P* < 0.05), the nucleus of Nrf2 and GCLC were upregulated by 1.06-fold and 2.59-fold (*P* < 0.05), and the expression of GSH in the experimental group was higher than that in the control group by 1.59 times (*P* < 0.05). After the combined use of arsenic with p38 inhibitor, the level of GCLC in combined experimental group was lower than that in the arsenic group (*P* < 0.05), these result show that arsenic can upregulate GCLC by activating p38/Nrf2. However, they found that arsenic promoted the expression of GCLC mainly through the activation of Nrf2 by ERK and JNK during acute exposure, while the content of p38 did not change. In addition, Thompson et al. [16] exposed the rat TAMH liver parenchyma cells after silencing Nrf2 to arsenic, it was found that arsenic can still promote GCLC expression by activating p38, indicating that Nrf2 is not involved in the regulation of GCLC by arsenic through p38. Therefore, whether arsenic can regulate GCLC through p38/Nrf2 still needs to be further clarify, our study will conduct a meta-analysis based on the experimental results of arsenic on GSH synthesis and reveal the effect of arsenic on GSH synthesis in vivo and in vitro.

## 2. Materials and Methods

This meta-analysis is based on the preferred reporting items for systematic reviews and meta-analysis (PRISMA).

### 2.1. Search Strategy

In this study, a total of 7 databases were retrieved, including PubMed, Cochrane Library, Web of Science, Excerpta Medica database (Embase), China National Knowledge Infrastructure (CNKI), Wan Fang Data databases, and SinoMed data, and the retrieval time of each database was from database construction to January 20, 2020.

The key words mainly included the following: Arsenic, Arsenite, ATO, As_2_O_3_, NaAsO_2_, glutamates, glutamic acid, glutamate, Glu, glusae, glutamic, l-glu, L(+)-Cysteine, Cys, 2-amino-3-mercaptopropanoic acid, C, cysh, l-beta-mercaptoalanine, cystine free base crystalline, cysteine, glycine, glycoconj, Gly, aminoacetic acid, *γ*-GCS, GCLC, GCLM, *γ*-glutamylcysteine synthetase, *γ*-glutamylcysteine synthetase heavy subunit chain, *γ*-glutamylcysteine synthetase light subunit chain, *γ*-glutamate cysteine ligase catalytic subunit, *γ*-glutamate cysteine ligase regulatory subunit, MAPK, p38, mitogen activated protein kinases, Nrf2, NF-E2, NF-E2-related factor2, glutathione, and GSH. Taking PubMed as an example, the specific retrieval formula is as follows: ((((((((arsenic) OR arsenite) OR ATO) OR As2O3) OR NaAsO2)) AND (((((((((((((((((((((((((((((glutamates) OR glutamic acid) OR glutamate) OR glutamic acid) OR Glu) OR glusa e) OR glutamic) OR l-glu) OR “L(+)-Cysteine”) OR Cys) OR cys) OR 2-amino-3-mercaptopropanoic acid) OR C) OR cysh) OR l-beta-mercaptoalanine) OR l cystine free base crystalline) OR cysteine) OR glycine) OR glycoconj) OR Gly) OR aminoacetic acid) OR “*γ*-GCS”) OR “GCLC”) OR “GCLM”) OR “*γ*-glutamylcysteine synthetase”) OR “r-glutamylcysteine synthetase heavy subunit chain”) OR “*γ*-glutamylcysteine synthetase light subunit chain”) OR “*γ*-glutamate cysteine ligase catalytic subunit”) OR “*γ*-glutamate cysteine ligase regulatory subunit”)) AND ((((((mapk) OR p38) OR mitogen activated protein kinases) OR Nrf2) OR “NF-E2”) OR “NF-E2-related factor2”)) AND ((glutathione) OR GSH)).

### 2.2. Inclusion Criteria

GSH plays an important role in inhibiting arsenic toxicity. In order to further clarify the effect of arsenic on GSH synthesis, we will formulate inclusion criteria according to PICO principle in this study.

#### 2.2.1. Type of Study

The experimental studies are published in Chinese and English.

#### 2.2.2. Participants

In vivo experiment, the object of study is human, mouse, rabbit, and so on. In vitro experiment, the subject was cell from normal or cancerous tissue.

#### 2.2.3. Intervention

The experimental group was poisoned with arsenic and arsenic compounds, such as NaAsO_2_, As_2_O_3_, and other forms of arsenic. If the longest time and maximum dose of GSH in the study were different from other indexes, it was consistent with the time and dose of GSH.

#### 2.2.4. Comparison

The blank control group did not receive any treatment.

#### 2.2.5. Outcome

GSH, Glu, Cys, Gly, GCLC, GCLM, p38, p-p38, and Nrf2 were described by $\bar{X}\pm S$.

### 2.3. Exclusion Standard

#### 2.3.1. Repeated Article

Repeated article includes repeated publication in Chinese and English, repeated collection between databases, and data publication of the same author using the same research object and method.

#### 2.3.2. Form of Publication

A form of publication includes the following: books, because authors may use this part of the original data to publish articles, in order to prevent duplicate entry; academic conference reports: there were two reasons to exclude it, firstly, academic conference report data may lack in mean or standard deviation, and secondly, some authors may use this part of the original data to publish articles, and in order to prevent repeated entry, we do not include the academic conference report.

#### 2.3.3. Subjects

Subjects include nonhuman, nonmouse, nonrabbit, and other animals such as shrimp, plants, and microorganisms.

#### 2.3.4. Research Methods

Research methods include nonexperimental studies such as meta-analysis and systematic review.

#### 2.3.5. Incomplete Data

Incomplete data include the lack of mean or standard deviation or inability to extract mean or standard deviation data from a chart, or lack of control group.

### 2.4. Search Results

**Figure 1:**
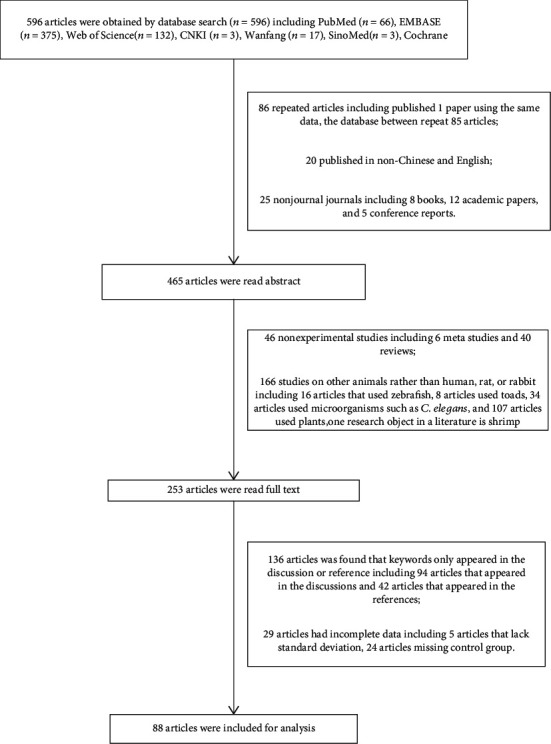
Search process and results.

According to the search strategy, a total of 596 articles were searched, and a total of 88 articles were included according to the inclusion and exclusion criteria for data analysis. Our search strategy was performed by two investigators using the same keywords independently. The screening results are shown in Figure 1. A total of 596 articles were included based on the title and keywords of the articles. A total of 131 articles were excluded according to the inclusion exclusion criteria. It mainly included 86 repeated articles, 20 published in non-Chinese and English, 25 nonjournal journals including 8 books, 12 academic papers, and 5 conference reports. 465 articles were retained, and 211 articles were found to meet the exclusion criteria by reading abstracts, including 46 nonexperimental studies including 6 meta-analysis studies and 40 reviews, 166 studies on other animals rather than human, rat, or rabbit including 16 articles that used zebrafish, 8 articles that used toads, 34 articles that used microorganisms such as *C. elegans*, and 109 articles that used plants, and one research object in a literature is shrimp. Subsequently reading 253 articles, it was found that the keywords only appeared in the discussion or reference, 136 articles including 94 articles appeared in the discussions, and 42 articles appeared in the references. In addition, it was also found that 29 articles had incomplete data including 5 articles that lack standard deviation and 24 articles missing a control group. In the end, a total of 88 articles were included for analysis.

### 2.5. Risk of Bias within Individual Studies

#### 2.5.1. Quality evaluation

The Cochrane risk offset quality assessment tool in Review Manger 5.3 was used for quality assessment, which mainly included (1) random sequence generation (selection bias), (2) the allocation concealment selection bias, (3) blinding of participants and personnel (performance bias), (4) blinding of outcome assessment (detection bias), (5) incomplete outcome data (attrition bias), and (6) selective reporting (reporting bias).

### 2.6. Risk of Bias across Studies

#### 2.6.1. Publication Bias

We used a funnel plot to evaluate whether there was a publication bias in the included articles.

#### 2.6.2. Sensitivity Analysis

The Stata 12.0 software was used for sensitivity analysis. The Chi-square test was performed with *α* = 0.05 as the significance level; when *P* < 0.05, the difference was considered statistically significant.

### 2.7. Statistical Analysis

This study will investigate the effects of arsenic on Glu, Cys, p38, p-p38, Nrf2, GCLC, GCLM, and GSH in vivo and in vitro and further explain the effect of arsenic on glutathione synthesis.

We mainly used the Review Manger 5.3 and Stata 12.0 software for data analysis. All the original data were recorded using mean ± standard deviation. Due to a large number of study in vivo and in vitro, the description units of arsenic exposure dose exists in many different ways (mg/kg, *μ*g/L, *μ*mol/L, etc.), and the results are bigger; we used the standardized mean difference (SMD) for the combined effect description so that it would more effectively combine the results of the research. SMD is mainly used to describe data that has the same purpose but cannot be directly compared. The formula is $d_{i}={\bar{X}}_{1i}-{\bar{X}}_{2i}/S_{i}$, *i* = 1, 2, 3, ⋯*k*. The combined effect amount of SMD was hypothetically tested at the significance level of *α* = 0.05; if *P* ≤ 0.05, the combined effect amount is considered statistically significant. Heterogeneity is divided into two degrees according to *I*^2^, *I*^2^ < 50% is low heterogeneity that is acceptable, *I*^2^ ≥ 50% is high heterogeneity, and *α* = 0.05 was used as the significance level for hypothesis testing of heterogeneity *I*^2^. When *P* < 0.05, *I*^2^ ≥ 50%, indicating heterogeneity among multiple studies, the combined effect of SMD and its 95% confidence interval is estimated by the random effect model, and when *P* > 0.05, *I*^2^ < 50%, indicating homogeneity among multiple studies, the fixed effect model was used to estimate the combined effect and its 95% confidence interval. When heterogeneity exists, a subgroup analysis should be conducted to find the cause of heterogeneity, and the combined effect of the experimental group and the control group was described by SMD and its 95% confidence interval.

## 3. Result

### 3.1. Basic Features of Included Literature

A total of 88 articles were included in this study, including 36 in vivo experiments and 52 in vitro experiments. The basic characteristics of the included articles are shown in Tables 1 and 2. In in vivo and in vitro experiments, the types of arsenic poisoning in the experimental group included sodium arsenite (NaAsO_2_) and arsenic trioxide (As_2_O_3_). The control group was blank control without any treatment. In the subgroup analysis, due to different exposure time of arsenic, the experimental exposure time in vivo was divided into <72 h or ≥72 h, and the exposure time in vitro was divided into <24 h or ≥24 h. Due to the different exposure doses of arsenic, the experimental exposure dose in vivo was divided into <10 mg/kg or ≥10 mg/kg, and the exposure dose in vitro was divided into ≤10 *μ*mol/L or >10 *μ*mol/L. According to the report, the relationship between dosage or reaction time and the research of arsenic exposure type will affect the synthesis of GSH [17]. The type of arsenic exposure in the body was divided into NaAsO_2_ or As_2_O_3_ or other, and the in vitro experiment was divided into NaAsO_2_ or As_2_O_3_. The research indicators include GSH, Glu, Cys, Gly, glutathione synthesis rate-limiting enzyme subunit GCLC and GCLM, and GCLC-regulated pathway indicators p38, p-p38, and Nrf2.

### 3.2. Quality Evaluation

**Figure 2:**
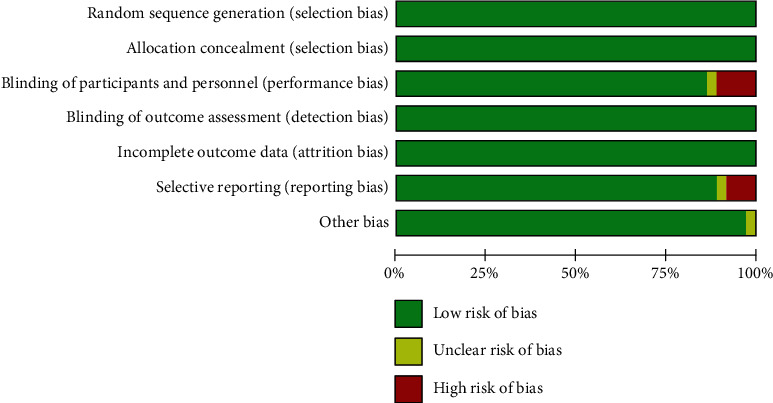
In vivo experiment quality evaluation results. This study included 36 articles with a low risk rate of more than 75 percent.

**Figure 3:**
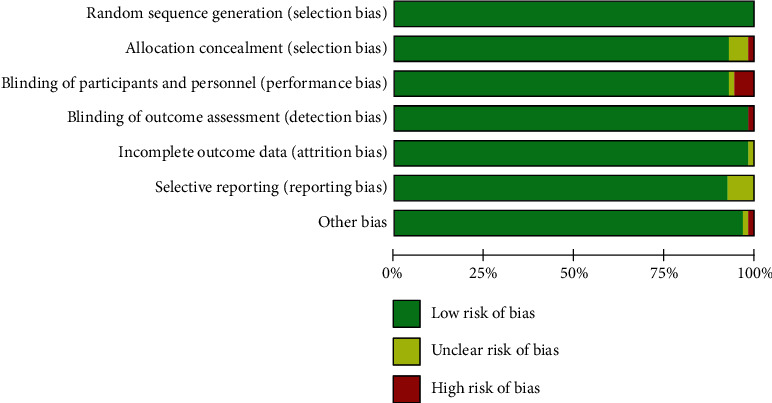
In vitro experiment quality evaluation results. This study included 52 articles with a low risk rate of more than 75 percent.

In vivo and in vitro studies were performed separately for quality evaluation. The quality of studies in vivo found that the low risk was over 75% and the high risk rate was only 13% (as shown in Figure 2); the in vitro study had a low risk rate of over 75% and a high risk rate of only 10% (Figure 3).

### 3.3. Meta-Analysis of Arsenic on GSH

**Figure 4:**
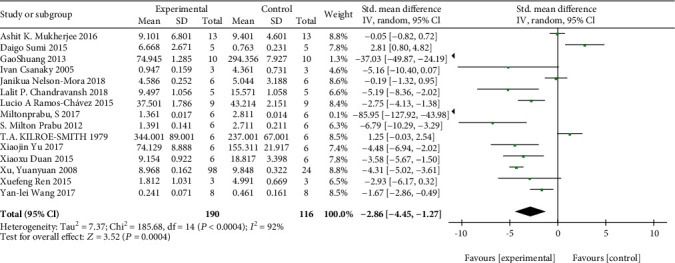
Meta-analysis of the effects of arsenic exposure on GSH in vivo. The forest plot shows the effect of arsenic treatment on GSH in the experiment and control group. SMD: standardized mean difference; IV: independent variable; 95% CI: 95% confidence interval; SD: standard deviation. The *P* value of the overall test effect is 0.00001; when *P* < 0.05, the difference was considered statistically significant.

**Figure 5:**
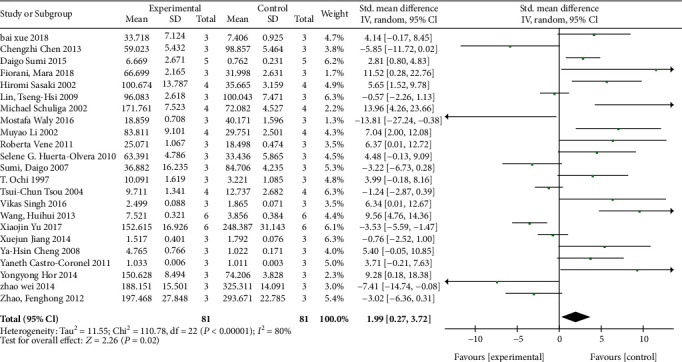
Meta-analysis of the effects of arsenic exposure on GSH in vitro. The forest plot shows the effect of arsenic treatment on GSH in experiment and control group. SMD: standardized mean difference; IV: independent variable; 95% CI: 95% confidence interval; SD: standard deviation. The *P* value of the overall test effect is 0.02; when *P* < 0.05, the difference was considered statistically significant.

The effects of arsenic on GSH were investigated in vivo and in vitro. In vivo studies showed that arsenic exposure reduced GSH content compared with the control group (SMD = −2.86, 95% CI (-4.45, -1.27)). But the results showed that compared with the control group, the GSH content was increased in arsenic exposed cells in vitro (SMD = 1.99, 95% CI (0.27, 3.72)). This result indicated that arsenic exposure can reduce GSH synthesis in vivo, but in vitro experiment of arsenic exposure can increase GSH synthesis (Figures 4 and 5).

### 3.4. Meta-Analysis of the Effect of Arsenic on Glu, Cys, and Gly

**Figure 6:**
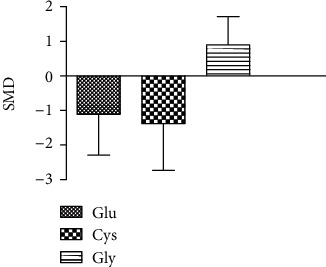
Meta-analysis of the effects of arsenic on Glu, Cys, and Gly in vivo. SMD; standardized mean difference. The *P* value of the Glu's overall effect test is 0.04. The *P* value of the Cys's overall effect test is 0.01. The *P* value of the Gly's overall effect test is 0.36. When *P* < 0.05, the difference was considered statistically significant.

**Figure 7:**
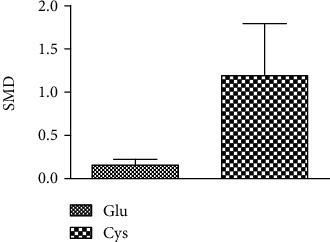
Meta-analysis of the effects of arsenic on Glu, Cys, and Gly in vitro. SMD: standardized mean difference. The *P* value of the Glu's overall effect test is 0.96. The *P* value of the Cys's overall effect test is 0.19. When *P* < 0.05, the difference was considered statistically significant.

According to the meta-analysis of the effects of arsenic on Glu, Cys, and Gly in vivo and in vitro (Figures 6 and 7), the results of arsenic exposure could decrease Glu (SMD = −1.11, 95% CI(-2.20, -0.02)) and Cys (SMD = −1.48, 95% CI (-2.63, -0.33)) and increase Gly (SMD = 0.79, 95% CI (-0.91, 1.49)) compared with the control group in vivo. This indicates that in in vivo experiments, arsenic exposure can inhibit GSH synthesis by reducing Glu and Cys content.

### 3.5. Effects of Arsenic on the p38/Nrf2 Pathway of GSH Synthesis Rate-Limiting Enzyme Subunit GCLC

**Figure 8:**
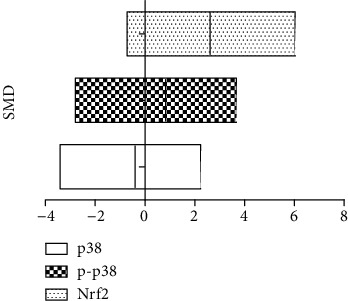
Meta-analysis of the effects of arsenic on p38, p-p38, and Nrf2 in vivo. SMD: standardized mean difference. Both ends of the segment represent the upper and lower limits of 95% CI, and the length of the segment represents the 95% CI range. When the 95% CI range contains 0, the difference is not statistically significant.

**Figure 9:**
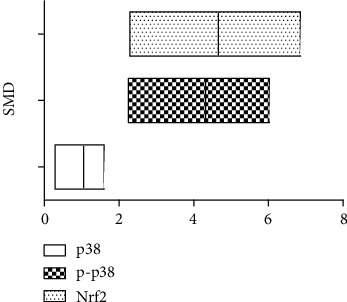
Meta-analysis of the effects of arsenic on p38, p-p38, and Nrf2 in vitro. SMD: standardized mean difference. Both ends of the segment represent the upper and lower limits of 95% CI, and the length of the segment represents the 95% CI range. When the 95% CI range contains 0, the difference is not statistically significant.

**Figure 10:**
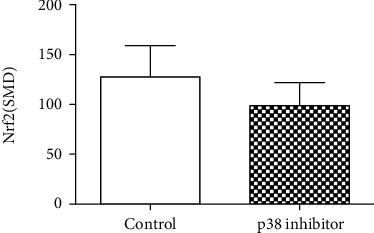
Meta-analysis of the effect of p38 inhibitor on Nrf2 in vitro. SMD: standardized mean difference. The *P* value of the overall effect test is 0.04. When *P* < 0.05, the difference was considered statistically significant.

**Figure 11:**
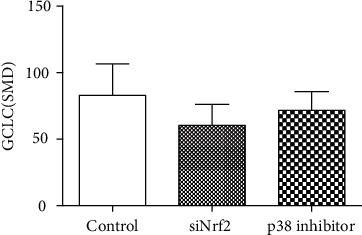
Meta-analysis of the effects of siNrf2 and p38 inhibitor on GCLC in vitro. SMD: standardized mean difference. Compared with control, the *P* value of the siNrf2 group's overall effect test is 0.02, and the *P* value of the p38 inhibitor group's overall effect test is 0.00001. When *P* < 0.05, the difference was considered statistically significant.

Studies have shown that arsenic exposure can act on the p38/Nrf2 pathway to change the expression level of GSH synthesis rate-limiting enzyme subunit GCLC thereby regulating the synthesis of GSH. According to the meta-analysis of the effects of arsenic on p38, p-p38, and Nrf2 (Figures 8 and 9), in vitro studies suggest that arsenic exposure can activate p38 (SMD = 0.93, 95% CI (0.35, 1.51)), promote phosphorylation of p38 (SMD = 4.19, 95% CI (2.34, 6.05)), and then promote Nrf2 entry into the nucleus (SMD = 4.60, 95% CI (2.34, 6.86)). In addition, we found that when p38 inhibitor was used (Figure 10), the expression of Nrf2 (SMD = −1.27, 95% CI (-2.46, -0.09)) in the nucleus decreased, the expression level of GCLC (SMD = −5.37, 95% CI (-5.37, -2.20)) decreased significantly, and the expression of GCLC (SMD = −2.12, 95% CI (-3.96, -0.28) also decreased when Nrf2 was only silenced (Figure 11). This indicates that in in vitro experiments, arsenic exposure promotes GSH synthesis and activates the p38/Nrf2 pathway.

### 3.6. Effects of Arsenic on the Subunits GCLC and GCLM of Glutathione Synthesis Rate-Limiting Enzymes

**Figure 12:**
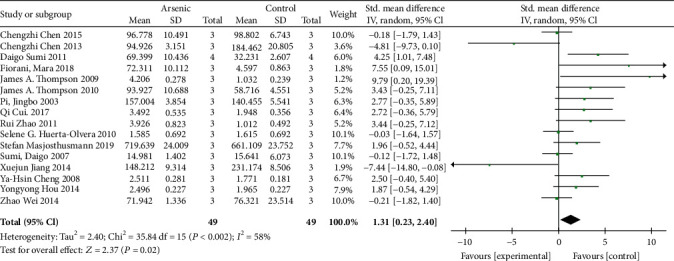
Meta-analysis of the effect of arsenic on the GCLC of r-GCS subunits in vitro. The forest plot shows the effect of arsenic treatment on GSH in the experiment and control group. SMD: standardized mean difference; IV: independent variable; 95% CI: 95% confidence interval; SD: standard deviation. The *P* value of the overall test effect is 0.02; when *P* < 0.05, the difference was considered statistically significant.

**Figure 13:**
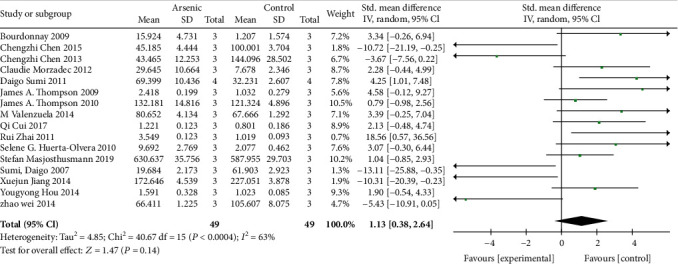
Meta-analysis of the effect of arsenic on the GCLM of r-GCS subunits in vitro. The forest plot shows the effect of arsenic treatment on GSH in the experiment and control group. SMD: standardized mean difference; IV: independent variable; 95% CI: 95% confidence interval; SD: standard deviation. The *P* value of the overall test effect is 0.14; when *P* < 0.05, the difference was considered statistically significant.

**Figure 14:**
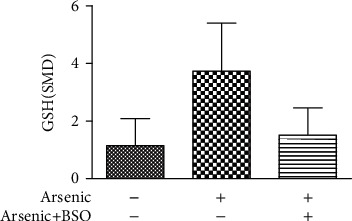
Effect of in vitro arsenic combined with r-GCS inhibitor on GSH. SMD: standardized mean difference. Compared with control, the *P* value of the arsenic group's overall effect test was <0.001, and the *P* value of the arsenic+BSO group's overall effect test was <0.001. When *P* < 0.05, the difference was considered statistically significant.

**Figure 15:**
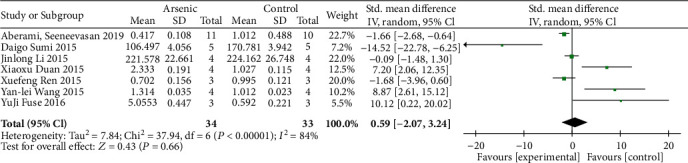
Meta-analysis of the effect of arsenic on the GCLC of r-GCS subunits in vivo. The forest plot shows the effect of arsenic treatment on GSH in the experiment and control group. SMD: standardized mean difference; IV: independent variable; 95% CI: 95% confidence interval; SD: standard deviation. The *P* value of the overall test effect is 0.66; when *P* < 0.05, the difference was considered statistically significant.

**Figure 16:**
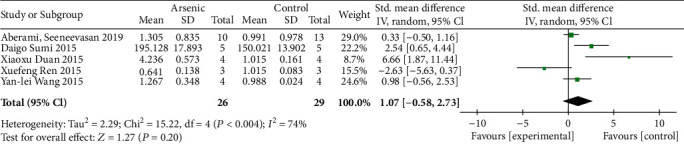
Meta-analysis of the effect of arsenic on the GCLM of r-GCS subunits in vivo. The forest plot shows the effect of arsenic treatment on GSH in the experiment and control group. SMD: standardized mean difference; IV: independent variable; 95% CI: 95% confidence interval; SD: standard deviation. The *P* value of the overall test effect is 0.20; when *P* <0.05, the difference was considered statistically significant.

According to the meta-analysis of the effects of arsenic on GCLC and GCLM in vitro (Figures 12 and 13), the effect of arsenic exposure on GCLM was not statistically significant, but it could promote the expression of catalytic subunit GCLC (SMD = 1.32, 95% CI (0.23, 2.40)), and we found that GSH synthesis was reduced when a combination of glutathione synthetase inhibitors was used (Figure 14). However, the results of meta-analysis in vivo (Figures 15 and 16) showed that the effects of arsenic exposure on GCLC and GCLM were not statistically significant. This result indicates that arsenic exposure promotes the expression of catalytic subunit GCLC during the process of increasing GSH synthesis in vitro.

### 3.7. Dose-Dependent Relationship between the Effects of Arsenic Exposure on GSH In Vitro

**Figure 17:**
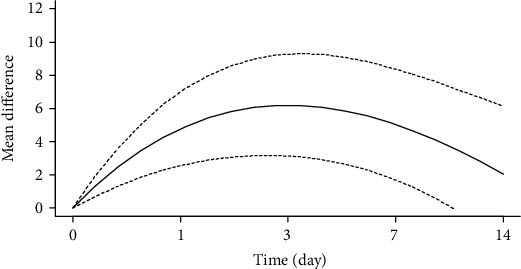
Dose-response relationship of arsenic exposure dose to GSH in vitro.

We used the spline model to investigate the effect of arsenic exposure time on GSH (Figure 17). The results showed that when the arsenic exposure dose is less than 10 *μ*mol/L, and the GSH content increases significantly as the arsenic exposure dose increases (SMD_5*μ*mol/L_ = 19.59, 95% CI (12.58, 26.93), SMD_10*μ*mol/L_ = 25.67, 95% CI (15.83, 35.68)), but when the arsenic exposure dose is greater than 10 *μ*mol/L, the GSH content no longer increases (SMD_20*μ*mol/L_ = 10.25, 95% CI (20.76, 31.76)). This indicates that the effect of arsenic exposure on GSH is double-sided.

### 3.8. Subgroup Analysis of Arsenic Exposure Doses In Vivo

**Figure 18:**
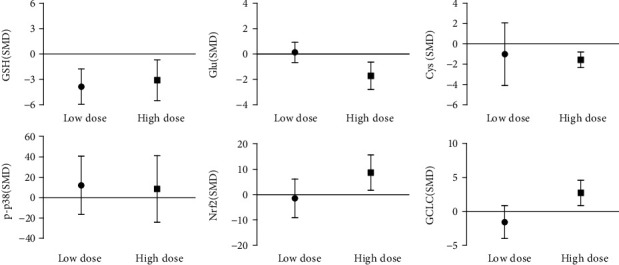
Subgroup analysis of arsenic exposure doses in vivo. SMD: standardized mean difference. Both ends of the line segment represent the upper and lower limits of 95% CI, and the length of the line segment represents the 95% CI range. When the 95% CI range contains 0, the difference is not statistically significant compared with the control group.

The in vivo arsenic exposure dose subgroup analysis results show that the high dose (>10 mg/kg) of arsenic exposure decreased the content of GSH and Glu and Cys, 3.06 times and 1.54-fold and 2.05-fold, compared with the control group. In addition to high dose of arsenic exposure on p-p38, influence has no statistical significance, but the expression of Nrf2 and GCLC was promoted by 5.13 times and 2.50 times. However, the study found that compared with the control group, low-dose arsenic exposure only reduced the GSH content by 3.38 times (Figure 18). This indicates that high dose of arsenic exposure can inhibit glutathione synthesis by promoting Nrf2 and GCLC and decreasing the content of Cys and Glu.

### 3.9. Subgroup Analysis of Arsenic Exposure Doses In Vitro

**Figure 19:**
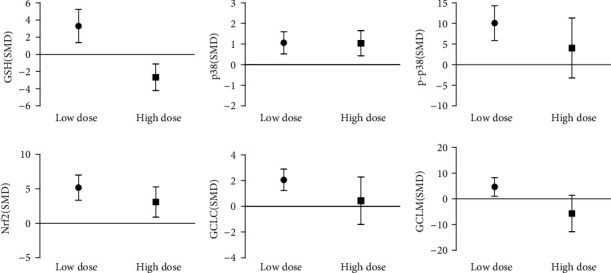
Subgroup analysis of arsenic exposure doses in vitro. SMD: standardized mean difference. Both ends of the line segment represent the upper and lower limits of 95% CI, and the length of the line segment represents the 95% CI range. When the 95% CI range contains 0, the difference is not statistically significant compared with the control group.

In vitro subgroup analysis of arsenic exposure dose showed that the GSH content in the low-dose group (≤10 *μ*mol/L) was 3.32 times higher than that in the control group, and p38, p-p38, Nrf2, GCLC, and GCLM were upregulated by 0.97 times, 3.94 times, 4.26 times, 2.03 times, and 2.25 times. However, the study found that compared with the control group, high-dose arsenic exposure reduced the GSH content by 2.16 times and increased the expression levels of p38 and Nrf2 by 1.41 times and 3.88 times, respectively, but exposure on p-p38, GCLM, and GCLC were not statistically significant (Figure 19). This indicates that low-dose arsenic exposure in vitro can promote GSH synthesis by promoting GCLC expression of p38/Nrf2.

### 3.10. Subgroup Analysis of Arsenic Exposure Time In Vivo

**Figure 20:**
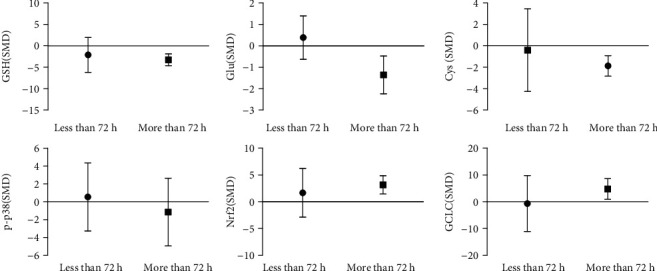
Subgroup analysis of arsenic exposure time in vivo. SMD: standardized mean difference. Both ends of the line segment represent the upper and lower limits of 95% CI, and the length of the line segment represents the 95% CI range. When the 95% CI range contains 0, the difference is not statistically significant compared with the control group.

In vivo analysis of arsenic exposure time subgroup (as shown in Figure 20) showed that long-term arsenic treatment decreases GSH content by 2.09 times, and Glu and Cys decreased by 1.37 times and 1.89 times, respectively, compared with the control group. Although the high doses of arsenic exposure on p-p38 had no statistically significant, the expression levels of Nrf2 and GCLC were 3.00 times and 2.38 times, respectively, higher than that of the control group. And we found that acute treatment of arsenic (<72 h) effect on GSH, Glu, Cys, p-p38, Nrf2, and GCLC had no statistically significant. These results indicate that long-term arsenic exposure in vivo inhibits GSH synthesis by inhibiting Cys and Glu and promoting Nrf2 and GCLC expression.

### 3.11. Subgroup Analysis of Arsenic Exposure Time In Vitro

**Figure 21:**
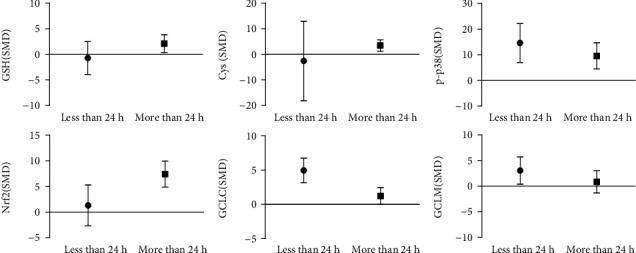
Subgroup analysis of arsenic exposure time in vitro. SMD: standardized mean difference. Both ends of the line segment represent the upper and lower limits of 95% CI, and the length of the line segment represents the 95% CI range. When the 95% CI range contains 0, the difference is not statistically significant compared with the control group.

Arsenic exposure time analysis in vitro showed that acute arsenic treatment increased the p-p38 expression by 14.4 times and increased GCLC and GCLM by 4.95 times and 3.34 times, respectively, but for GSH, Cys, and Nrf2 there was no statistically significant difference. In addition, chronic arsenic exposure (≥24 h) increased GSH and Cys levels by 2.37 times and 1.46 times, respectively, and increased p-p38, Nrf2, and GCLC expression levels by 9.54 times, 6.22 times, and 1.26 times, respectively (as Figure 21). These results suggest that chronic arsenic exposure can promote GSH synthesis by increasing intracellular Cys content and activating p38/Nrf2 to promote GCLC expression in vitro.

### 3.12. Subgroup Analysis of Arsenic Exposure Species In Vivo

**Figure 22:**
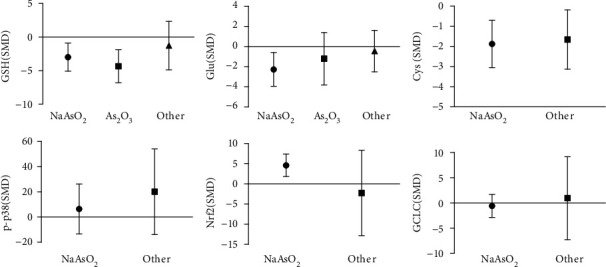
Subgroup analysis of arsenic exposure species in vivo. SMD: standardized mean difference. Both ends of the line segment represent the upper and lower limits of 95% CI, and the length of the line segment represents the 95% CI range. When the 95% CI range contains 0, the difference is not statistically significant compared with control group.

Subgroup analysis of arsenic exposure species in vivo (Figure 22) showed that NaAsO_2_ exposure decreased the content of GSH (SMD = −3.17, 95% CI (-5.05, -1.30)), Glu (SMD = −2.08, 95% CI (-3.63, -0.53), and Cys (SMD = −1.78, 95% CI (-3.34, -0.21)) and promoted the expression of Nrf2 (SMD = 4.51, 95% CI (0.16, 8.87)). The As_2_O_3_ exposure decreased the content of GSH (SMD = −4.48, 95% CI (-6.94, -2.02)). This result indicates that NaAsO_2_ exposure inhibits GSH synthesis decreasing the content of Glu and Cys and promoting Nrf2 expression in vivo.

### 3.13. Subgroup Analysis of Arsenic Exposure Species In Vitro

**Figure 23:**
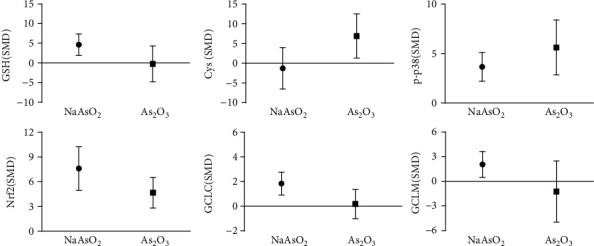
Subgroup analysis of arsenic exposure species in vitro. SMD: standardized mean difference. Both ends of the line segment represent the upper and lower limits of 95% CI, and the length of the line segment represents the 95% CI range. When the 95% CI range contains 0, the difference is not statistically significant compared with the control group.

The subgroup analysis of arsenic exposure species in vitro (as shown in Figure 23) showed that NaAsO_2_ exposure increased the expression of GSH (SMD = 3.19, 95% CI (0.89, 5.49)), p-p38 (SMD = 3.83, 95% CI (1.95, 5.71)), Nrf2 (SMD = 6.08, 95% CI (2.01, 10.16)), GCLC (SMD = 2.14, 95% CI (0.80, 3.47)), and GCLM (SMD = 1.64, 95% CI (0.31, 2.96)). In addition, As_2_O_3_ exposure increased the content of Cys (SMD = 6.18, 95% CI (0.28, 12.07)) and promoted the expression of Nrf2 (SMD = 4.10, 95% CI (0.74, 7.47)) and p-p38 (SMD = 5.34, 95% CI (1.54, 9.14)). This result indicates that NaAsO_2_ exposure promoted GSH synthesis by promoting GCLC and GCLM expression by p38/Nrf2, but As_2_O_3_ exposure inhibited GSH synthesis by promoting Nrf2 and p-p38 and decreased the content of Cys.

### 3.14. Publication Bias Analysis

**Figure 24:**
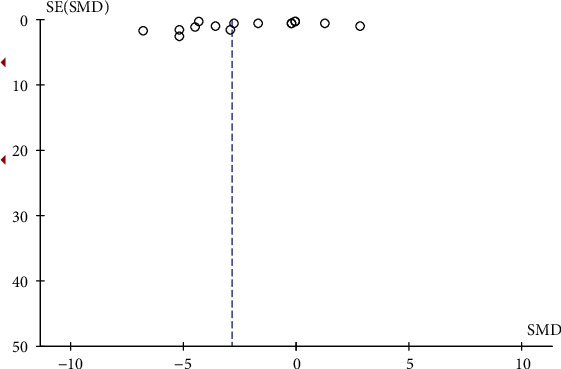
In vivo experiment published biased funnel chart. SMD: standardized mean difference. SE: standard error.

**Figure 25:**
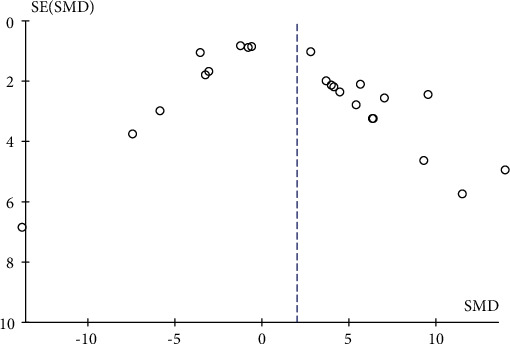
In vitro experiment published biased funnel chart. SMD: standardized mean difference. SE: standard error.

According to the funnel plot (as shown in Figures 24 and 25), all studies in the in vivo and in vitro experiments were symmetrically arranged around the center line, indicating that there was no publication bias in both in vivo and in vitro experiments.

### 3.15. Sensitivity Analysis

**Figure 26:**
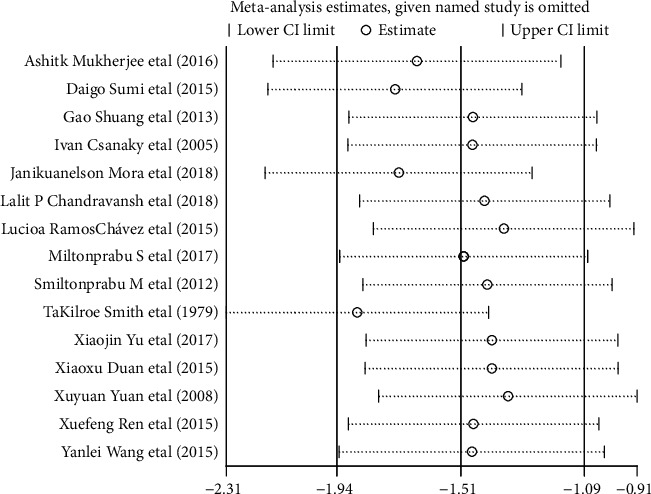
Sensitivity analysis of the effect of arsenic on GSH in vivo.

**Figure 27:**
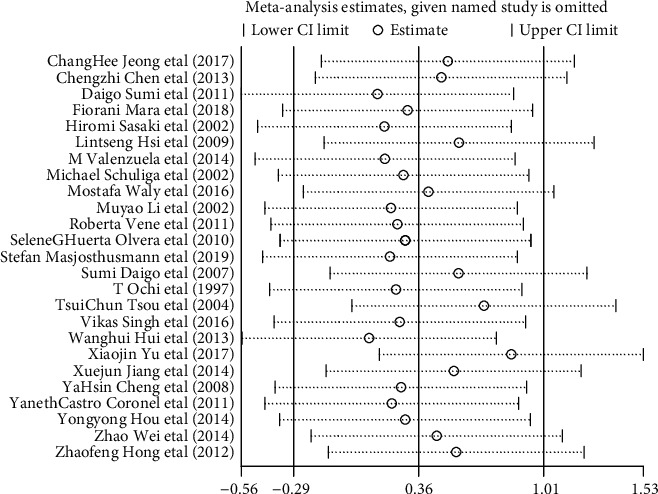
Sensitivity analysis of the effect of arsenic on GSH in vitro.

Sensitivity analysis was performed by taking the action of arsenic on GSH as an example in vivo and in vitro (Figures 26 and 27). According to the results, if any of the studies were removed, the effect amount was located around the overall predicted value and did not exceed the upper and lower limits of the overall predicted value of 95% CI. In addition, the 95% CI on-line after removing any of the poststudy effects was lower than the overall predictive effect, and the 95% CI on-line was higher than the overall predictive effect. The above result shows that after the exclusion of a single study in vivo and in vitro, the effect on the overall result is small, the sensitivity is low, and the overall result is relatively stable.

## 4. Discussion

A large number of studies indicates that GSH plays an important role in the inhibition of oxidative stress by arsenic. However, there are still many controversies about the effects of arsenic on GSH synthesis. We conducted a meta-analysis of the effects of arsenic exposure on GSH synthesis in vivo and vitro. The results showed that arsenic exposure inhibited GSH synthesis by reducing intracellular Glu and Cys content in vivo, while arsenic exposure promoted GSH synthesis by activating p38/Nrf2 in vitro. These results provide a theoretical basis for revealing the mechanism of arsenic on GSH synthesis.

GSH is synthesized by Glu, Cys, and Gly. Excitatory amino acid transporters (EAAT) and metabolite transporters are important transporters for maintaining intracellular Glu levels. Nelson et al. [18] have shown that arsenic exposure can reduce intracellular Glu level by inhibiting EAAT1 expression. The cystine/glutamate reverse transporter (X^−^_c_ system) is one of the important Cys transporters [19], Wang et al. [20] found that low-dose arsenic exposure can activate Nrf2 to promote X^−^_c_ system expression and increase intracellular Cys levels thereby promoting GSH synthesis. The meta-analysis of in vivo experiment found that chronic and high-dose arsenic exposure could promote the expression of *γ*-GCS subunit GCLC by upregulating Nrf2, but GSH synthesis is still reduced, which may be due to the decrease in the content of GSH synthesis substrate Cys, so the intracellular content of Glu and Cys determines the amount of GSH synthesis. When Glu and Cys enter the cell, *γ*-glutamylcysteine is synthesized under the catalysis of *γ*-GCS, and then GSH is synthesized [9]. However, studies have shown that when GSH is oversynthesised, it reversely acts on *γ*-GCS, and inhibits *γ*-GCS activity to maintain intracellular GSH balance [21]. Therefore, *γ*-GCS plays an important role in regulating GSH synthesis. *γ*-GCS is mainly composed of heavy chain GCLC and light chain GCLM. GCLC is the main functional subunit involved in catalyzing the binding of Glu and Cys. Studies have shown that arsenic exposure promotes the expression of GCLC by promoting the entry of Nrf2 into the nucleus, promoting the combination of Nrf2 with the original ARE containing the GCLC target gene promoter [22]. Sumi et al. [23] also found that arsenic exposure could modulate GCLC transcription by activating Nrf2. In this study, we demonstrated that chronic, high-dose arsenic exposure increased GCLC expression by promoting Nrf2 expression. At the same time, we found that GCLC expression was also decreased when Nrf2 was silenced. In addition, when GCLC was inhibited by the *γ*-GCS inhibitor BSO, the synthesis of GSH is reduced. Therefore, Nrf2 plays an important role in the regulation of GSH synthesis by arsenic through GCLC, Glu, and Cys.

**Figure 28:**
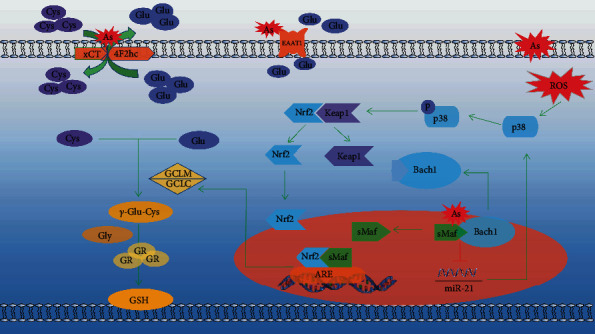
The mechanism of arsenic influence on GSH synthesis.

Nrf2 is an important transcription factor to regulate antioxidant response. Under physiological conditions, Nrf2 binds closely to Keap1 in the cytoplasm. When oxidative stress Nrf2/Keap1 is separated by ubiquitination, Nrf2 enters into the nucleus and binds to sMaf, further identifying and binding with ARE [24, 25]. Wang et al. [26] found that phosphorylation of p38 can act on Nrf2/Keap1 to cause ubiquitination and dissociation, thereby promoting Nrf2 entry into the nucleus. Duan et al. [13] showed that arsenic exposure produces a large amount of ROS, which can promote the expression of p38, Nrf2, and GCLC. In this study, we also found that low-dose chronic, arsenic exposure promoted p38 phosphorylation and promoted nuclear transcription of Nrf2 to promote GCLC expression in vitro. In addition, we found that p38 inhibitor reduced the expression of Nrf2 when the expression of p38 decreased, as well as the expression level of GCLC, indicating that p38/Nrf2 played an important role in the regulation of GSH synthesis by arsenic through GCLC. However, we found that arsenic had no significant effect on p-p38 when exposed to high doses, but it still promoted the expression of Nrf2 and GCLC. This indicates that other signaling molecules may be involved in the regulation of GCLC through p38/Nrf2 by arsenic. Bach1 is a nuclear transcriptional repressor molecule that binds to sMaf in the nucleus and further competitively binds ARE to Nrf2. Liu et al. [27] showed that arsenic exposure could promote the export of Bach1 from the nucleus to the cytoplasm and promote the entry of Nrf2 into the nucleus to bind with ARE. In addition, Mansoor et al. [28] found that the expression of miR-21 significantly decreased after silencing Bach1 gene with siRNA. Chen et al. [29] reported that p38 expression decreased when miR-21 was overexpressed, and Lucia et al. [30] found that when the expression level of miR-21 was increased, the expression level of Nrf2 significantly decreased. Therefore, As Figure 28 shows, Bach1 and miR-21 may play an important role in the process of arsenic regulating GCLC and mediating GSH synthesis by p38/Nrf2.

## 5. Conclusions

The above results suggest that arsenic exposure has a certain regulatory effect on glutathione synthesis, but these results also show the difference between in vivo and in vitro studies of the effect of arsenic on glutathione synthesis. In in vivo study, arsenic exposure can reduce GSH synthesis by reducing intracellular glutamate and cysteine levels. However, in in vitro experiments, chronic low-dose arsenic exposure can activate the p38/Nrf2 pathway, upregulate GCLC expression, and promote glutathione synthesis. This study clarifies the influence factors of arsenic on glutathione synthesis and provides a direction for further research on the effect of arsenic on glutathione synthesis.

### 5.1. Limitations and Perspectives

The research from two aspects of in vivo and in vitro analyses of arsenic for GSH synthesis influence still have considerable heterogeneity in in vivo experiments of GCLC, p38. In future research, we will continue to conduct experiments on cells, animals, and humans validated with the result of the study. In addition, some studies have shown that Bach1 and miR-21 may play an important role in the process of arsenic regulation of GSH synthesis by p38/Nrf2. Therefore, in the future, we will continue to study the role of miR-21 and Bach1 in arsenic-regulated GCLC through p38/Nrf2 to further improve the mechanism of arsenic on the synthesis of GSH.

## Figures and Tables

**Table 1:** Basic characteristics of the articles in vivo experiments.

Author	Year	*n*	Type of arsenic	Dose (mg/kg)	Time (h)	Outcome
Nelson et al. [31]	2018	6	NaAsO_2_	>10	≥72	GSH -0.19 [-1.32, 0.95], Glu -1.05 [-2.26, 0.22]
Wang et al. [20]	2015	8	As_4_S_4_	>10	≥72	GSH -1.67 [-2.86, -0.49], Glu -3.29 [-4.93, -1.65] Cys-2.91 [-4.43, -1.39], GCLC 8.87 [2.61, 15.12] GCLM 0.98 [-0.56, 2.53], Nrf2 0.12 [-1.27, 1.51]
Yu et al. [32]	2017	6	As_2_O_3_	≤10	≥72	GSH -4.48 [-6.94, -2.02]
Singh et al. [33]	2016	3	NaAsO_2_	≤10	≥72	Glu 6.76 [0.04, 13.48], Cys -0.71 [-2.46, 1.03]
Duan et al. [13]	2015	6	NaAsO_2_	>10	<72	GSH -3.58 [-5.67, -1.50], GCLC 7.20 [2.06, 12.35] GCLM 6.66 [1.87, 11.44], Nrf2 17.57 [5.41, 29.74]
Ramos-Chávez et al. [18]	2015	9	NaAsO_2_	>10	≥72	GSH -2.75 [-4.13,-1.38]
Prabu et al. [34]	2012	6	NaAsO_2_	≤10	≥72	GSH -6.79 [-10.29, -3.29], Nrf2 7.04 [3.42, 10.66]
Ren et al. [35]	2015	3	NaAsO_2_	>10	≥72	GSH -2.93 [-6.17, 0.32], GCLC -1.68 [-3.96, 0.60] GCLM -2.63 [-5.63, 0.37]
Gao et al. [36]	2013	10	NaAsO_2_	>10	≥72	GSH -37.03 [-49.87, -24.19], Nrf27.74 [4.92, 10.55]
Miltonprabu et al. [37]	2017	6	Na_3_AsO_4_	≤10	≥72	GSH -89.95 [-127.92, -43.98], Nrf2 -4.48 [-6.94, -2.02]
Sung et al. [38]	2019	5	NaAsO_2_	>10	≥72	Glu -4.82 [-7.80, -1.84]
Lu et al. [39]	2018	50	As_2_O_3_	≤10	≥72	Glu 0.56 [0.16, 0.96]
Huo et al. [40]	2016	7	As_2_S_2_	>10	≥72	Glu 0.27 [-0.78, 1.33], Cys -1.34 [-2.54, -0.14], Gly 3.28 [1.51, 5.06]
Shoufang et al. [41]	2014	14	NaAsO_2_	>10	≥72	Glu -2.07 [-3.01, -1.13]
Bei et al. [42]	2012	12	As_2_S_4_	>10	≥72	Glu -6.92 [-9.20, -4.63]
Huo et al. [43]	2012	8	As_4_S_4_	>10	≥72	Glu -3.86 [-5.69, -2.04], Gly -1.24 [-2.34, -0.14]
Sumi et al. [44]	2015	7	NaAsO_2_	≤10	≥72	GSH 2.81 [0.80, 4.82], GCLC -14.52 [-22.78, -6.25] GCLM 2.54 [0.65, 4.44], Nrf2 3.58 [1.22, 5.95]
Mukherjee et al. [45]	2016	193	NaAsO_2_	≤10	≥72	GSH -0.05 [-0.25, 0.15], Cys -2.38 [-2.64, -2.12]
Kilroe-Smith and McLoughlin [46]	1979	98	Na_2_HAsO_4_.7H_2_0	>10	<72	GSH 1.25 [-0.03, 2.54], Glu -0.60 [-1.70, 0.57] Gly 0.03 [-1.10, 1.16]
Fuse et al. [47]	2016	3	NaAsO_2_	≤10	<72	GCLC 10.12 [0.22, 20.02]
Li et al. [48]	2015	4	NaAsO_2_	>10	≥72	GCLC -0.09 [-1.48, 1.30], Nrf2 3.63 [0.78, 6.49]
Ivan and Zoltan [49]	2005	3	NaAsO_2_	≤10	<72	GSH -5.16 [-10.40, 0.07]
Nagaraja et al. [50]	1993	6	As_2_O_3_	≤10	≥72	Glu -6.84 [-10.37, -3.31]
Wang et al. [22]	2019	4	As_2_O_3_	≤10	<72	Glu 1.43 [-0.27, 3.13]
Aberami et al. [51]	2019	11	NaAsO_2_	≤10	<72	GCLC -1.66 [-2.66, -0.64], GCLM 0.33 [-0.50, 1.16]
Yi et al. [52]	2018	5	As_4_S_4_	>10	≥72	Glu 6.19 [2.49, 9.89]
Huang et al. [53]	2012	10	As_4_S_4_	>10	≥72	Glu 1.53 [0.50, 2.55]
Uthus et al. [54]	1990	15	Na_2_HAsO_4_.7H_2_0	≤10	≥72	Glu -0.17 [-1.15, 0.81], Cys 0.04 [-0.94, 1.02] Gly 1.46 [0.32, 2.60]
Zhao et al. [55]	2019	10	NaAsO_2_	≤10	≥72	p38 2.24 [1.07, 3.41] p-p38 0.67 [-0.24, 1.58]
Sun et al. [56]	2019	3	As_2_O_3_	>10	<72	p38 0.57 [-1.12, 2.27] p-p38 30.07 [0.99, 59.14]
Chandravanshi et al. [57]	2018	9	NaAsO_2_	≤10	≥72	GSH -5.19, [-8.36, -2.02], p-p38 1.30 [-0.14, 2.74]
Wei et al. [58]	2018	5	NaAsO_2_	≤10	<72	p38 -171 [-3.28, -0.14] p-p38 34.41 [15.00, 53.82]
Li et al. [59]	2017	3	NaAsO_2_	>10	<72	p38 0.32 [-1.31, 1.95] p-p38 1.45 [-0.68, 3.57]
Huang et al. [60]	2017	10	NaAsO_2_	>10	≥72	p38 -5.80 [-7.99, -3.61] p-p38 -5.80 [-7.99, -3.61]
Xu et al. [61]	2008	9	NaAsO_2_	≤10	≥72	GSH -4.31 [-5.02, -3.61]
Srivastava et al. [62]	2015	6	As_2_O_3_	≤10	<72	Nrf2 -14.71 [-21.98, -7.44]

*n* represents the number of parallel samples in the experimental group; GSH is a reducing molecule; Glu, Cys, and Gly are the prerequisites for GSH synthesis; GCLC is the heavy chain subunit of GSH synthesis rate-limiting enzyme *γ*-GCS; GCLM is the light chain subunit of GSH synthesis rate-limiting enzyme *γ*-GCS; p38 is a protein kinase; p-p38 represents phosphorylation of p38; and Nrf2 is a nuclear transcription factor.

**Table 2:** Basic characteristics of the articles in vitro experiments.

Author	Year	*n*	Type of arsenic	Dose (*μ*mol/L)	Time (h)	Outcome
Xiaojin Yu [32]	2017	6	As_2_O_3_	≤10	<24	GSH -3.53 [-5.59, -1.47], Nrf2 -0.52 [-1.68, 0.64]
Vikas Singh [33]	2016	3	NaAsO_2_	≤10	≥24	GSH 6.34 [0.01, 12.67], Glu 3.60 [-0.23, 7.43] Cys -5.82 [-11.66, 0.02], Nrf2 4.83 [-0.10, 9.75]
Yongyong Hou [15]	2014	3	NaAsO_2_	≤10	≥24	GSH 9.20 [0.10, 18.38], GCLC 1.87 [-0.54, 4.26] GCLM 1.90 [-0.54, 4.33], p38 -0.28 [-1.91, 1.34] p-p38 6.28 [0.01, 12.55], Nrf2 13.38 [0.36, 26.39]
Rui Zhao [63]	2011	3	As_2_O_3_	≤10	≥24	GCLC 3.44 [-0.25, 7.12], GCLM 18.56 [0.57, 36.56], Nrf2 21.91 [0.69, 43.12]
YanethCastro Coronel [64]	2011	3	NaAsO_2_	≤10	≥24	GSH 3.71 [-0.21, 7.63], Nrf2 5.21 [-0.07, 1048]
SeleneGHuerta Olvera [65]	2010	3	NaAsO_2_	>10	≥24	GSH 4.48 [-0.13, 9.09], GCLC -0.03 [-1.61, 1.57], GCLM 3.08 [-0.30, 6.44], Nrf2 8.29 [0.13, 16.45]
TsuiChun Tsou [9]	2004	4	NaAsO_2_	>10	≥24	GSH -1.24 [-2.87, 0.39]
Hiromi Sasaki [12]	2002	4	NaAsO_2_	≤10	<24	GSH 5.65 [1.52, 9.78], Cys 3.40 [0.68, 6.12]
Muyao Li [14]	2002	4	NaAsO_2_	≤10	≥24	GSH 7.04 [2.00, 12.08]
T Ochi [66]	1997	3	NaAsO_2_	≤10	≥24	GSH 3.99 [0.18, 8.16], Cys 0.29 [-1.33, 1.92]
Xuejun Jiang [67]	2014	3	As_2_O_3_	>10	≥24	GSH -0.76 [-2.52, 1.00], GCLC -7.44 [-14.80, -0.08] GCLM -10.31 [-20.39, -0.23], Nrf2 3.23 [-0.28, 6.73]
Wanghui Hui [68]	2013	6	NaAsO_2_	≤10	≥24	GSH 9.56 [4.76, 14.36], p38 -1.29 [-2.58, 0.01] p-p38 2.82 [1.04, 4.61], Nrf2 23.93 [12.19, 35.66]
Fiorani Mara [69]	2018	3	NaAsO_2_	≤10	<24	GSH 11.52 [0.28, 22.76], GCLC 7.55 [0.09, 15.01] Nrf2 3.42 [-0.25, 7.08]
Zhaofeng Hong [70]	2012	3	NaAsO_2_	>10	≥24	GSH -3.02 [-6.36, 0.31], Glu -4.57 [-7.07, -2.07]
Lintseng Hsi [71]	2009	3	NaAsO_2_	≤10	<24	GSH -0.57 [-2.26, 1.13]
Sumi Daigo [23]	2007	3	As_2_O_3_	>10	≥24	GSH -3.22 [-6.73, 0.28], GCLC -0.12 [-1.72, 1.48] GCLM-13.11 [-25.88, -0.35], Nrf213.31 [0.36, 26.26]
Pi Jingbo [72]	2003	3	NaAsO_2_	≤10	≥24	GCLC 2.77 [-0.35, 5.88], Nrf2 0.08 [-1.52, 1.68]
Zhao Wei [73]	2014	3	As_2_O_3_	≤10	≥24	GSH -7.41 [-14.74, -0.08], GCLC -0.21 [-1.82, 1.40], GCLM -5.43 [-10.91, 0.05], Nrf2 2.60 [-0.38, 5.57]
Irawan Susanto [74]	1998	3	NaAsO_2_	≤10	≥24	Cys 1.11 [-0.82, 3.03]
Daigo Sumi [75]	2011	4	NaAsO_2_	≤10	<24	GSH 2.81 [0.80, 4.83], GCLC 4.25 [1.01, 7.48], GCLM 4.25 [1.01, 7.48]
Chengzhi Chen [76]	2013	3	NaAsO_2_	>10	≥24	GSH -5.85 [-11.72, 0.02], GCLC -4.81 [-9.73, 0.10], GCLM -3.67 [-7.56, 0.22]
JamesA Thompson [77]	2010	3	NaAsO_2_	>10	≥24	GCLC 3.43 [-0.25, 7.11], GCLM 0.79 [-0.98, 2.56]
JamesA Thompson [16]	2009	3	NaAsO_2_	≤10	<24	GCLC 9.81 [0.20, 19.42], GCLM 4.58 [-0.12, 9.27], p38 1.46 [-0.67, 3.59], p-p38 12.58 [0.33, 24.83]
YaHsin Cheng [17]	2008	3	NaAsO_2_	≤10	≥24	GSH 5.40 [-0.05, 10.95], GCLC 2.55 [-0.39, 5.48]
Mostafa Waly [78]	2016	3	NaAsO_2_	≤10	<24	GSH -13.81 [-27.24, -0.38], Cys -9.08 [-17.99, -0.17]
Roberta Vene [79]	2011	3	As_2_O_3_	≤10	≥24	GSH 6.37 [0.01, 12.72], Cys 12.14 [0.31, 23.97]
Yan Wang [80]	2012	3	NaAsO_2_	≤10	≥24	Glu 1.66 [-0.61, 3.92]
Qi Cui [81]	2017	3	NaAsO_2_	≤10	≥24	GCLC 2.72 [-0.36, 5.79], GCLM 2.12 [-0.48, 4.72]
M Valenzuela [19]	2014	3	As_2_O_3_	≤10	≥24	GSH 2.06 [-0.49, 4.61], GCLM 3.39 [-0.257.04] Nrf2 9.88 [0.21, 19.55]
Claudie Morzadec [82]	2012	3	As_2_O_3_	≤10	<24	GCLM 2.28 [-0.44, 4.99]
Emilie Bourdonnay [83]	2009	3	As_2_O_3_	≤10	≥24	GCLM 3.34 [-0.26, 6.94], Nrf2 7.17 [0.06, 14.27]
Stefan Masjosthusmann [84]	2019	11	NaAsO_2_	≤10	≥24	GCLC 1.96 [-0.52, 4.44]1, GCLM 1.04 [-0.85, 2.93]
Chengzhi Chen [85]	2015	3	As_2_O_3_	>10	≥24	GCLC -0.18 [-1.79, 1.43], GCLM -10.72 [-21.19, -0.25]
SusanM Deneke [86]	1992	4	As_2_O_3_	≤10	≥24	Cys 4.66 [1.17, 8.15]
Michael Schuliga [87]	2002	4	NaAsO_2_	≤10	≥24	GSH 13.96 [4.26, 23.66], Cys 2.40 [0.25, 4.56]
ZhiYuan Liu [88]	2019	3	NaAsO_2_	≤10	<24	p38 1.57 [-0.64, 3.77], p-p38 10.25 [0.22, 20.29]
Pattama Singhirunnusom [89]	2018	3	NaAsO_2_	>10	<24	p38 2.39 [-0.42, 5.20], p-p38 24.26 [0.78, 47.75]
Yan Xia [90]	2018	3	NaAsO_2_	≤10	≥24	p38 1.08 [-0.03, 2.99], p-p38 1.18 [-0.79, 3.14]
Sunbin Ling [91]	2017	3	As_2_O_3_	≤10	≥24	p38 0.84 [-0.90, 2.63], p-p38 0.84 [-0.96, 2.63]
ChangHee Jeong [92]	2017	5	NaAsO_2_	>10	<24	GSH 4.14 [-0.17, 8.45], p38 0.94 [-0.90, 2.79] p-p38 72.20 [2.46, 141.93]
Jingyi Zhang [93]	2017	3	As_2_O_3_	≤10	≥24	p38 1.26 [-0.75, 3.27], p-p38 25.860.84, 50.89]
Arulkumar Nagappan [94]	2017	3	NaAsO_2_	≤10	≥24	p38 1.74 [-0.58, 4.05], p-p38 4.21 [-0.15, 8.59]
Jiamin Mao [95]	2016	3	As_2_O_3_	≤10	≥24	p38 0.82 [-0.96, 2.61], p-p38 15.82 [0.46, 31.18]
Daigo Sumi [96]	2016	3	NaAsO_2_	≤10	≥24	p38 2.08 [-0.49, 4.61], p-p38 3.33 [-0.26, 6.93]
Xuezhong Gong [97]	2016	3	NaAsO_2_	≤10	≥24	p38 1.60 [-0.62, 3.83], p-p38 9.92 [0.21, 19.64]
SunMi Yun [98]	2016	3	As_2_O_3_	≤10	≥24	p-p38 16.58 [0.49, 32.66]
IreneAmigo Jiménez [99]	2016	3	As_2_O_3_	≤10	≥24	p-p38 6.75 [0.04, 13.46]
HongGyum Kim [100]	2016	3	As_2_O_3_	≤10	≥24	p38 4.30 [-0.15, 8.75], p-p38 23.85 [0.76, 46.93]
M Låg [101]	2016	3	As_2_O_3_	≤10	≥24	p38 2.88 [-0.33, 6.10], p-p38 -5.57 [-11.19, 0.04]
Lian Zhang [102]	2015	3	As_2_O_3_	≤10	≥24	p38 -0.54 [-2.22, 1.14], p-p38 -0.82 [-2.60, 0.97]
Xuezhong Gong [103]	2015	3	NaAsO_2_	≤10	<24	p38 1.18 [-0.78, 3.15], p-p38 3.03 [-0.30, 6.37]
HuiWen Chiu [104]	2015	3	As_2_O_3_	≤10	≥24	p38 2.45 [-0.41, 5.31], p-p38 6.87 [0.05, 13.69]

*n* represents the number of parallel samples in the experimental group; GSH is a reducing molecule; Glu, Cys, and Gly are the prerequisites for GSH synthesis; GCLC is the heavy chain subunit of GSH synthesis rate-limiting enzyme *γ*-GCS; GCLM is the light chain subunit of GSH synthesis rate-limiting enzyme *γ*-GCS; p38 is a protein kinase; p-p38 represents phosphorylation of p38; and Nrf2 is a nuclear transcription factor.

## Data Availability

The data used to support the findings of this study are available from the corresponding author upon request.
